# Study of the utilization of main crop straw resources in Southern China and its potential as a replacement for chemical fertilizers

**DOI:** 10.3389/fpls.2023.1172689

**Published:** 2024-01-05

**Authors:** Guiting Mu, Lifu Xu, Jiachun Zhang

**Affiliations:** ^1^ Guizhou Institute of Biology, Guizhou Academy of Sciences, Guiyang, China; ^2^ Guizhou Provincial Forest Resources and Environment Research Center, Guizhou University, Guiyang, China; ^3^ Guizhou Botanical Garden, Guizhou Academy of Sciences, Guiyang, China

**Keywords:** quantity of straw, nutrient, nitrogen, P_2_O_5_, K_2_O, chemical fertilizer

## Abstract

Although straw returning to the field (SRTTF) is conducive to promoting sustainable agricultural production and protecting the environment, straw resources are still wasted due to the lack of suitable straw-returning technology in southern China. Based on the statistical yearbook and a large number of studies, different methods were used to calculate the total straw resources and SRTTF potential, and differences in these methods were compared. The results indicate that the total amount of straw resources in southern China in 2021 was 3.35×10^8^ t. The nutrient content of K_2_O in the straw accounted for the highest proportion of total nutrient resources (63.66%), followed by N (26.88%) and P_2_O_5_ (9.46%). In theory, total SRTTF could replace almost all K_2_O and part of N and P_2_O_5_, indicating that the nutrient substitution potential of SRTTF was high. It is suggested that the SRTTF method be adopted in the middle and lower reaches of the Yangtze River, which mainly uses direct returning (DR) supplemented by indirect returning (IDR). In southeast China, straw returning is carried out by the combination of IDR and IR. In southwest China, straw returning is mainly carried out by IR and supplemented by MDR. This study will provide theoretical support for the government to formulate straw-returning policy.

## 1 Introduction

Since the 1980s, the total annual amount of agricultural fertilizer used in China has increased yearly. According to the China Environmental Statistics Yearbook, the total amount of agricultural fertilizer used increased from 12,694,000 tons in 1980 to 51,913,000 tons in 2021 (a 300-fold increase). In 2015, the total amount of agricultural fertilizer used was as high as 60,226,000 tons. Although the amount of fertilizer used has decreased since 2015, the total annual amount of fertilizer used is still over 5,000,000 tons. The application of fertilizers can promote crop growth and development, effectively increase crop yield, ensure food security, and play a crucial role in China’s poverty alleviation efforts (He et al., 2021; Van Wesenbeeck et al., 2021). However, the excessive application of fertilizers can have negative effects on the growth, yield, and quality of crops (Jiang et al., 2020; Zhang et al., 2023). Every step involved in the use of fertilizer—from production to application—can have deleterious effects on agriculture, human health, and the ecological environment (Schnug and Lottermoser, 2013; Sun et al., 2020). Some researchers have found that the products and by-products of fertilizers include NH_4_, CO_2_, CH_4_, toxic chemicals, and gases. Deliberate discharge of untreated solid, liquid, or gaseous waste poses a serious threat to the environment (Wang et al., 2020; Rathi et al., 2021). The excessive application of chemical fertilizers can lead to soil salinization, heavy metal accumulation, water eutrophication, nitrate accumulation, increases in greenhouse gas emissions, nitrogen and phosphorus runoff, and other problems (Wu et al., 2020; Liu, 2020; Roozbeh and Rajaie, 2021). Some researchers have found that workers in fertilizer production factories and the application of fertilizers by farmers are exposed to ionizing radiation from natural radioactive nuclides (238U, 232Th, and 210Po), which can increase the risk of cancer (Nyambura et al., 2020; Zhang et al., 2020; Gatti et al., 2022).

Various solutions have been proposed to address a series of environmental issues posing threats to agriculture and human health caused by the excessive use of fertilizers. Biofertilizers such as bacteria, Chinese herbal residues, and organic fertilizers rich in rock and plant substances can improve the yield and quality of crops, vegetables, and fruits such as rice, corn, lettuce, garlic, citrus, pineapple, grapes, and oil palm (Wu et al., 2021; Jin et al., 2022; Liang et al., 2022). The substitution of chemical fertilizer with biological fertilizer can reduce soil acidification; increase the soil organic matter content, soil active nitrogen content, nutrient content, and enzyme activity; improve soil physical and chemical properties and the microbial community; and enhance soil metabolism, which can reduce the adverse effects of fertilizer loss on the environment (Gao et al., 2022; Zhang et al., 2023). Agricultural and domestic wastes such as straw, rice husk, animal excrement, sludge, sediment https://doi.org/10.1007/s10098-021-02109-9, slag, kitchen waste, and urban garbage have been used to replace some fertilizers through composting or preparing biochar and modified biochar (Das et al., 2019; Cheng et al., 2023; Fang et al., 2022). Previous studies have found that this method can reduce greenhouse gas emissions, improve soil fertility and microbial community structure (Han et al., 2021; Zhang et al., 2021), promote crop growth, and increase yield (Dong et al., 2021; Yang et al., 2023). Some researchers focused on policy formulation and others have found that strict restrictions on the use of chemical fertilizers affect the profitability of planting systems and do not significantly improve environmental quality (Weerahewa and Dayananda, 2023). Increasing subsidies for manure, strengthening the services provided by agricultural cooperatives, expanding the scale of farms, increasing agricultural knowledge, promoting a sense of social responsibility among citizens, using e-commerce, increasing the consumption of agricultural products, and optimizing policies can provide incentives for farmers to make green planting decisions, thereby reducing the use of fertilizers and promoting the long-term sustainable development of agriculture (Tian et al., 2023).

The various aforementioned methods are aimed at reducing the use of fertilizers, and this is achieved through the use of biological organic matter, agricultural and industrial waste, and other raw materials to prepare organic fertilizer, which can be used as a replacement for chemical fertilizer; the use of organic fertilizer can alleviate a series of problems that affect agricultural and environmental safety associated with fertilizer application by reducing industrial production links, but this method is unable to fundamentally solve these problems. Yin et al. (2018) analyzed a large dataset on the annual crop yield, crop sown area, and fertilizer consumption of different crop types in 31 provinces of mainland China from 1998 to 2014. Analyses showed that straw returning to the field (SRTTF) could achieve balanced K_2_O, P_2_O_5_, and N in fertilizer; the results of this study indicate that straw resources could be used to completely replace chemical potassium fertilizer. However, specific technology for replacing fertilizers with straw resources has not been used in this study. Here, the total amount of straw resources in southern China was estimated based on statistical yearbooks and a large number of studies using different methods to assess the potential for straw resources to replace fertilizers in different regions. We also analyzed differences among assessment methods and identified straw nutrient return utilization methods that were optimal for different areas according to regional variation in environmental characteristics and planting patterns. The aim of our study was to explore the utility of an agricultural planting model in which biological resources completely replace fertilizers. This approach permits resources to be conserved, is environmentally friendly, improves agricultural land productivity, ensures food security, and promotes long-term environmental sustainability.

## 2 Research methods and data sources

### 2.1 Research area and regional division

**Figure 1:**
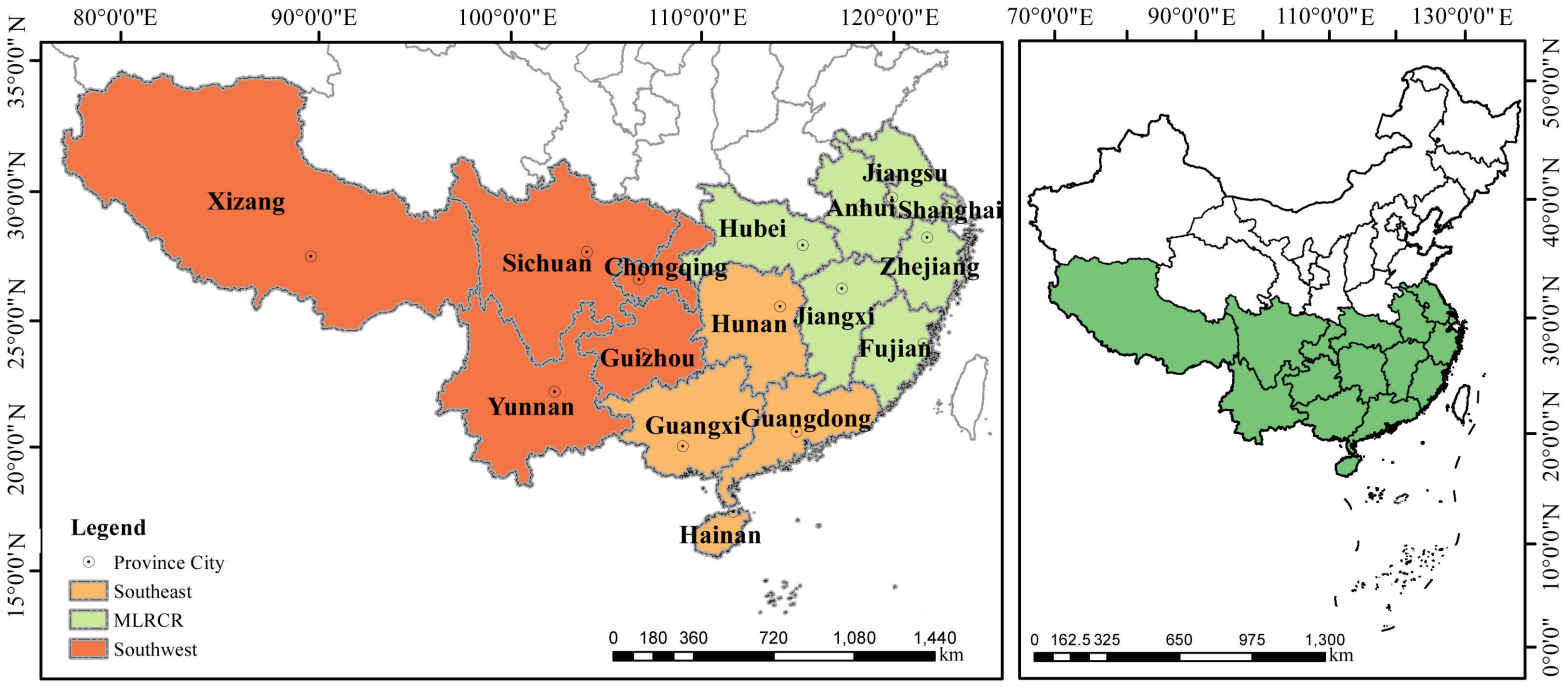
Overview map of the study area.

China is a vast area, with complex terrain, diverse soil types, and various climates; consequently, several planting methods are used. Compared with the northern regions dominated by plains, the southern regions of China (south of the Qinling Huaihe River and east of the Qinghai-Tibet Plateau) are mostly mountainous and hilly, and there is significant variation in altitude and planting systems in this region; thus, spatial heterogeneity is high, the level of mechanization is low, and the amount of straw resources is variable among areas in southern China (**Figure 1**). Therefore, the straw-returning techniques employed for the reuse of straw resources in southern China should be based on the environmental characteristics and local conditions of different agricultural regions. The southern region of China examined in this article includes 16 provinces, municipalities, and autonomous regions, with the exception of Hong Kong, Macao, Taiwan, and the South China Sea Islands. According to the division of China’s main grain-producing areas, southern China can be divided into three agricultural areas: the middle and lower reaches of the Yangtze River (MLRCR), including the seven provinces (municipalities) of Hubei, Hunan, Shanghai, Anhui, Jiangsu, Zhejiang, and Jiangxi; the southwest region, including Yunnan, Xizang, Chongqing, Sichuan, and Guizhou Provinces; and the Southeast region, including Guangxi, Fujian, Guangdong, and Hainan Provinces. Southern China can be divided into two cropping areas based on planting patterns: 1) Hainan, Guangxi, Guangdong, Fujian, Jiangxi, Hunan, and Zhejiang Provinces (autonomous regions), and 2) the rice-wheat growing area, including the Shanghai, Jiangsu, Hubei, and Anhui provinces (cities). There is one wheat-growing region in Tibet. The rice/corn-growing areas include the Sichuan, Guizhou, and Yunnan provinces and Chongqing. Rape, soybean, peanut, rice, corn, wheat, and potato are the main crops grown in South China. Therefore, the utility and applicability of these seven crop straw resources as replacements for chemical fertilizer were analyzed in this study.

### 2.2 Method for Quantifying Straw Nutrient Resources

Although a database of crop yield data was established in 1865, the management of crop straw did not receive much attention until 1970 (Johnson et al., 2006). No statistical analyses have been used to quantify crop straw resources (Gao et al., 2009). This paper employs a common method for calculating total straw (W_i_). Specifically, W_j_ was calculated as the ratio of economic crop yield to the quantity of straw resources (grass-grain ratio). The amount of nutrient resources was then determined according to the nutrient content of the straw (Cui et al., 2021; Wang et al., 2023). The formulas were as follows:


(1) $$W_{i}=\sum_{j=1}^{7}Y_{ij}\times R_{j}$$

(2) $$WN(N)=W_{j}\times N_{j}$$

(3) $$WP(P_{2}O_{5})=W_{j}\times P_{j}\times 2.29$$

(4) $$WK(K_{2}O)=W_{j}\times K_{j}\times 1.2$$

In Equations 1-4, W_i_ is the number of crop straw resources in the ith province, city, and autonomous region; Y_ij_ is the yield of the jth crop in the ith province, municipality, and autonomous region; R_j_ is the grass-grain ratio of the jth crop (Liu and Li, 2017; Wang et al., 2023) (**Table 1**); WN is the nutrient resource of straw nitrogen (N); W_j_ is the quantity of straw resources for the jth crop; N_j_ is the nitrogen content of the jth crop straw; WP is the nutrient content of straw phosphorus (P_2_O_5_); P_j_ is the phosphorus nutrient content of the jth crop straw; 2.29 is the coefficient for the conversion of simple phosphorus into phosphorus pentoxide (P_2_O_5_); WK is straw potassium (K_2_O) nutrient resources; K_j_ is the potassium nutrient content of the jth crop straw; and 1.2 is the coefficient of the conversion of potassium into potassium oxide (K_2_O), where i=1, 2, 3,…, 16, j=1, 2, 3, 4, 5, 7.

**Table 1:** Grass-to-Grain Ratio and Straw Nutrient Content in Southern China.

Crops	grass-grain ratio				Nutrient content in straw(%)		
	MLRCR	Southeast	Southwest	mean value	N	P_2_O_5_	K_2_O
Rice	1.08	0.95	0.93	0.99	0.82	0.13	1.9
Wheat	1.39	1.49	1.36	1.41	0.54	0.09	1.16
Corn	1.29	1.28	1.27	1.28	0.89	0.11	0.99
Soybean	1.41	1.69	1.53	1.54	0.89	0.09	0.64
Peanut	1.18	1.2	1.6	1.33	1.64	0.15	1.56
Rape	2.64	2.55	2.75	2.65	0.64	0.13	2.01
Potato	0.47	0.47	0.46	0.47	2.35	0.49	2.76

MLRCR (The middle and lower reaches of the Yangtze River).

### 2.3 Calculation of crop nutrient demand

The nutrient demand of crops is calculated based on the planting area of crops and the recommended fertilization amount. The data on crop planting area were sourced from the “China Rural Statistical Yearbook 2022,” and the recommended fertilization amount per unit area of crops was based on experimental data from the International Institute of Plant Nutrition (**Table 2**) (Li et al., 2017). The formula for calculating the nutrient demand of crops is as follows:

**Table 2:** The recommended fertilization amount per unit area for crops.

Crops	Recommendation(kg/hm^2^)		
	N	P_2_O_5_	K_2_O
Rice	162	62	96
Wheat	165	84	74
Corn	158	52	68
Soybean	70	89	96
Peanut	103	92	125
Rape	130	74	92
Potato	172	101	131


(5) $$N_{i}=\sum_{i=1}^{n}\frac{Aci\times Fci}{1000}$$

In Equation 5, N_i_ represents the nutrient demand of the ith crop (×10^4^ tons); i represents the ith crop, where i=1, 2, 3,…, 7; Aci represents the planting area of the ith crop (×10^4^hm^2^); and Fci represents the recommended fertilization amount per unit area for the ith crop (kg/hm^2^) (Li et al., 2017).

### 2.4 Method for estimating the fertilizer substitution potential of straw returning to the field

The amount of fertilizer that can be substituted with crop SRTTF was estimated based on the seasonal release of nutrients (ARN) per unit area sown (Liu and Li, 2017), and the formula is as follows:


(6) $$A_{RN}=\frac{W_{RN}}{A}=\frac{W_{N}\times R_{N}}{A}$$

(7) $$A_{RP}=\frac{W_{RP}}{A}=\frac{W_{P}\times R_{P}}{A}$$

(8) $$A_{RK}=\frac{W_{RK}}{A}=\frac{W_{K}\times R_{NK}}{A}$$

In Equations 6-8, A_RN_, A_RP_, and A_RK_ represent the nitrogen, phosphorus, and potassium nutrient resources available per unit area of crop SRTTF during the current season (representing the nutrient substitution potential); W_RN_, W_RP_, and W_RK_ represent the nitrogen, phosphorus, and potassium nutrient resources that can be provided during the season when crop straw is returned to the field (representing the substitutable amount of nutrients); R_N_, R_P_, and R_K_ are the release rates of nitrogen, phosphorus, and potassium during the season when straw is returned to the field (**Table 3**) (Liu and Li, 2017); and A is the crop planting area.

**Table 3:** Rate of in-season nutrient release from straw.

Crops	Rate of in-season nutrient release from straw %		
	N	P_2_O_5_	K_2_O
Rice	47.19	66.69	84.91
Wheat	50.11	62.01	89.05
Corn	54.04	73.03	84.43
Soybean	52.06	54.41	84.30
Peanut	51.61	66.50	85.82
Rape	52.65	66.31	82.18
Potato	–	81.00	76.90

### 2.5 Data sources

The total crop yield and crop planting area data in this study were obtained from the “China Rural Statistical Yearbook 2022” and “China Statistical Yearbook 2022.” Data on the grass-grain ratio and nitrogen, phosphorus, and potassium content in the calculations of crop straw resources (**Table 1**) were obtained from relevant studies.

### 2.6 Data processing

Statistical analysis of the data was mainly carried out in Microsoft Excel, and the map of the quantity of straw resources and the distribution of straw nutrient resources was obtained using ArcGIS Map10.1 software.

## 3 Results and recommendations

### 3.1 Main crop straw resources in Southern China

#### 3.1.1 Total amount of straw resources for major crops in southern China

**Table 4** can be obtained by substituting the total output of various crops, the grass-grain ratio in **Table 1**, and the nutrient content in straw into formulas 1 to 4. As shown in **Table 4**, the total amount of straw in southern China is 3.35×10^8^ tons, with rice, corn, wheat, and rapeseed accounting for 50.26%, 17.33%, 16.35%, and 9.99% of the total straw resources, respectively. The nutrient resources of the three major grain crops accounted for 55.26% (rice), 13.67% (corn), and 11.84% (wheat) of the total nutrient resources, and rape straw accounted for 11.34% of nutrient resources. K_2_O accounted for the highest proportion of total nutrient resources (63.66%), followed by N (26.88%) and P_2_O_5_ (9.46%). The N, P_2_O_5_, and K_2_O content in rice was the highest, which accounted for 49.61%, 51.20%, and 58.25% of the total nutrients, respectively.

**Table 4:** Nutrient resources contained in straw of different crops and their proportions in total straw in southern China in 2021.

Crops	The amount of straw		The nutrient content in straw					
	The total amount ×10^8^t	Proportion (%)	N ×10^4^t	Proportion (%)	P_2_O_5_ ×10^4^t	Proportion (%)	K_2_O ×10^4^t	Proportion (%)
Rice	1.68	50.26	131.95	49.61	47.90	51.20	366.88	58.24
Wheat	0.55	16.35	29.58	11.12	11.29	12.06	76.24	12.10
Corn	0.58	17.33	51.68	19.43	14.63	15.63	68.98	10.95
Soybean	0.07	2.18	6.51	2.45	1.51	1.61	5.61	0.89
Peanut	0.08	2.43	13.38	5.03	2.80	2.99	15.27	2.42
Rape	0.33	9.99	21.43	8.06	9.97	10.65	80.77	12.82
Potato	0.05	1.45	11.45	4.31	5.47	5.84	16.14	2.56
Total	3.35	100.00	265.96	100.00	93.56	100.00	629.89	100.00

#### 3.1.2 Spatial distribution of the resources of major crops in southern China

**Figure 2:**
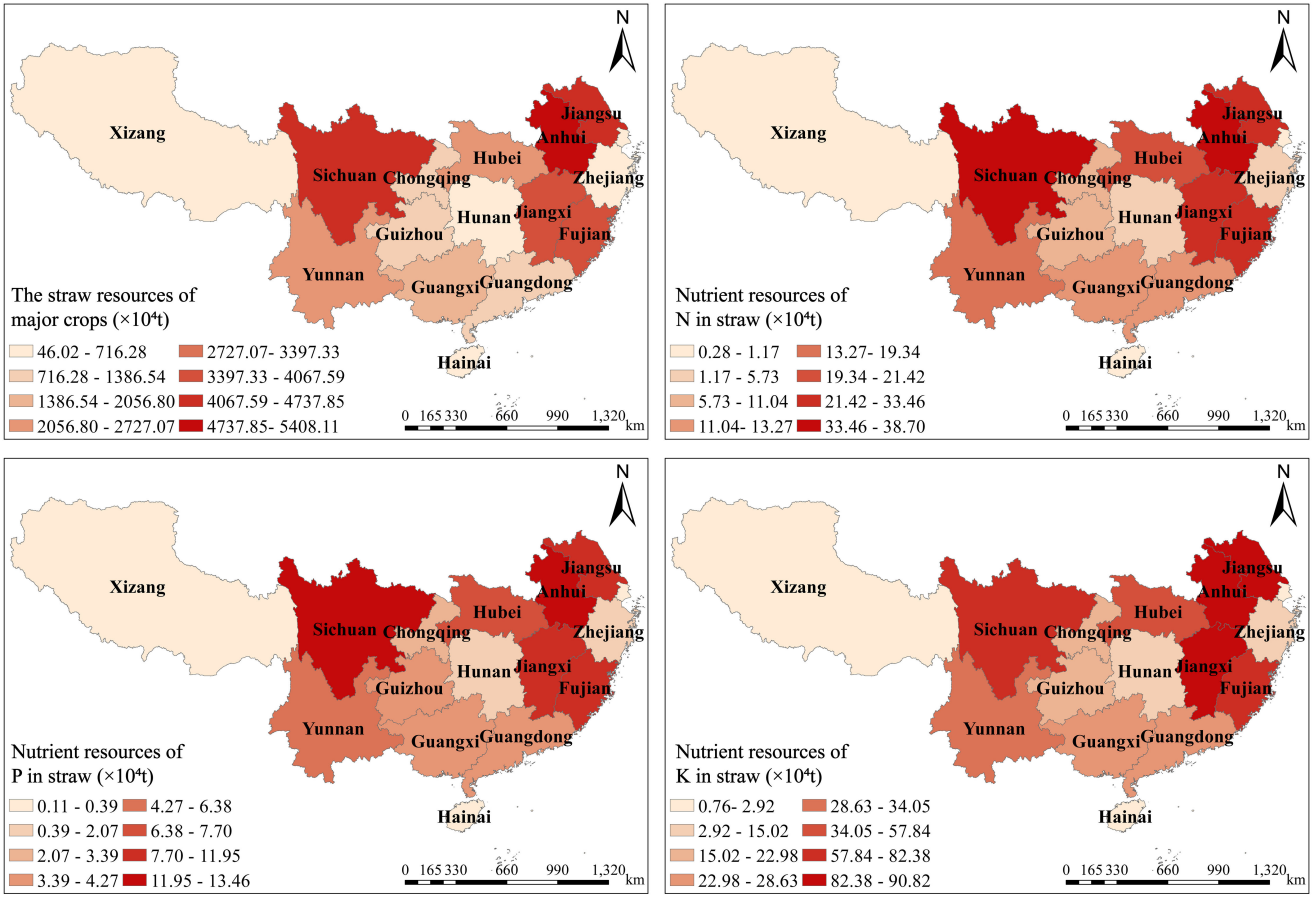
Distribution of straw resources of major crops in different regions of southern China in 2021.

**Figure 2** can be obtained by substituting the total output of various crops, the grass-grain ratio in **Table 1**, and the nutrient content in straw into formulas 1 to 4. Significant regional variation in the quantity of straw resources in southern China was observed in 2021. The total amount of straw resources in MLRCR was relatively large, accounting for 63.60% of the total amount in the southern region. The top three provinces in terms of straw resources were Anhui, Jiangsu, and Sichuan, which accounted for 16.19%, 13.96%, and 13.30% of the total straw resources in the south, respectively. The proportion of straw nutrient resources was highest in Anhui Province, followed by Sichuan Province and Jiangsu Province. Anhui, Sichuan, and Jiangsu Provinces accounted for 14.13%, 13.18%, and 12.83% of the total straw nutrient resources in the southern region, respectively. The total amount of straw resources was lowest in Shanghai (1.04×10^6^ t) and Xizang (0.46 ×10^6^). Straw production was greater than 3×10^7^t for the five provinces: two provinces had straw production between 2×10^7^t and 3×10^7^t; four provinces had straw production between 1×10^7^t and 2×10^7^t; four provinces had straw production between 1×10^6^t and 1×10^7^t, and only one province had straw production less than 1×10^6^t.

### 3.2 Nutrient demand of major crops in southern China’s provinces

**Figure 3:**
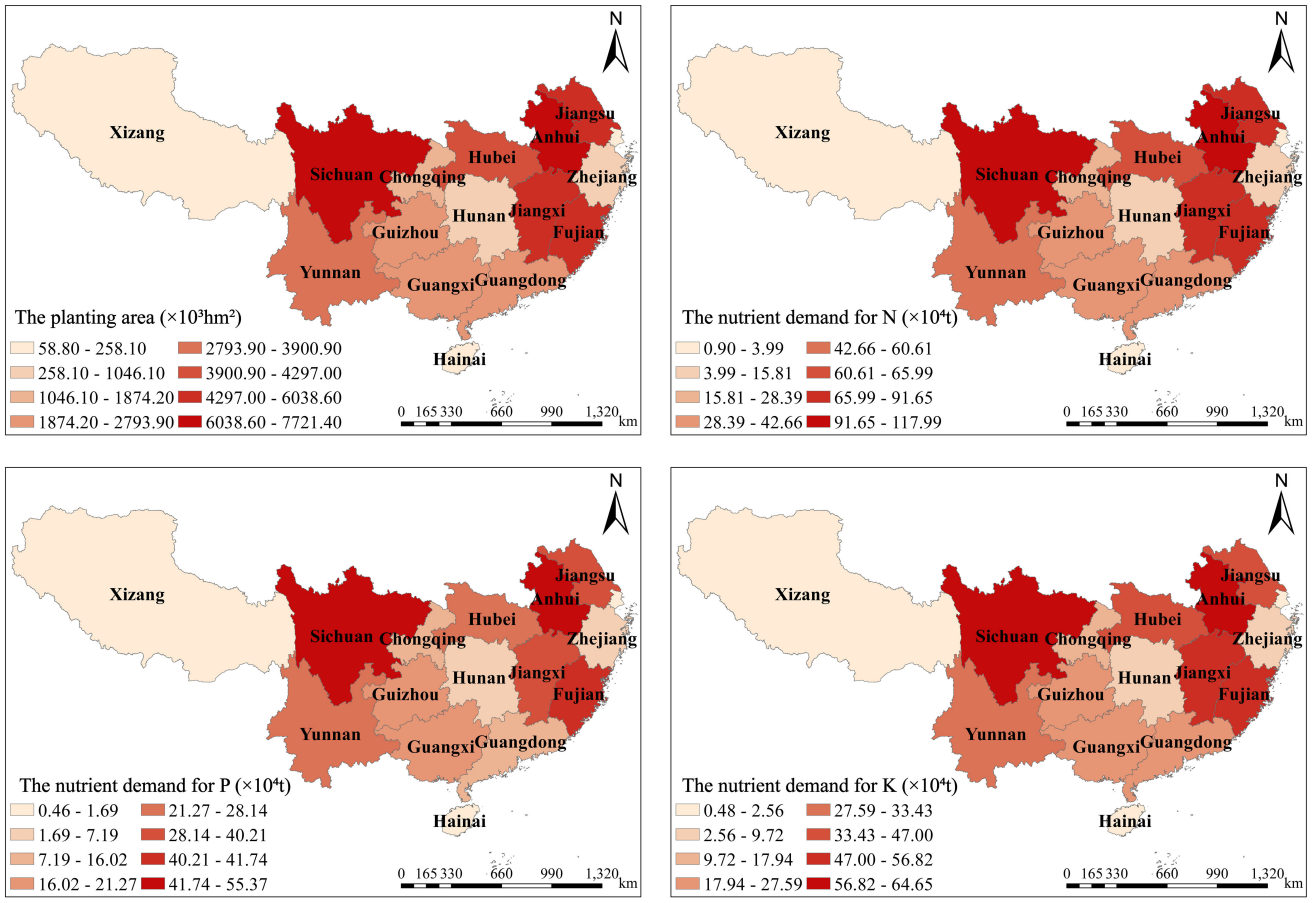
Nutrient demand and yield of main crops in provinces of southern China in 2021.

The nutrient requirements for major crops in southern China’s provinces were calculated by substituting the total yield of crops in each province, the grass-grain ratio, and nutrient content in straw in **Table 1** into formulas 1 to 4 (**Figure 3**). The planting area of rice, corn, wheat, potato, peanut, soybean, and rape in South China in 2021 was 5.3×10^7^hm^2^, and the theoretical demand for N, P_2_O_5_, and K_2_O of these seven crops was 8.01×10^6^t, 3.65×10^6^t, and 4.78×10^6^t, respectively. The total theoretical nutrient demand of the seven crops in MLRCR was 9.57×10^6^t, which accounted for 58.20% of the total theoretical nutrient demand of the seven crops in southern China, and the theoretical nutrient demand of N, P_2_O_5_, and K_2_O in this region was 4.70×10^6^t, 2.13×10^6^t, and 2.74×10^6^t, respectively. The theoretical demand for N in the various provinces in southern China ranged from 0.9×10^4^t to 117.99×10^4^t, the theoretical demand for P_2_O_5_ ranged from 0.46×10^4^t to 55.37×10^4^t, and the theoretical demand for K_2_O ranged from 0.48×10^4^t to 66.65×10^4^t. The theoretical demand for N was highest in Anhui Province, Sichuan Province, and Jiangxi Province, at 1.18×10^6^t, 1.04×10^6^t, and 0.92×10^6^t, respectively. Anhui Province, Sichuan Province, and Fujian Province had the highest theoretical demand for P_2_O_5_, at 0.55×10^6^t, 0.50×10^6^t, and 0.42×10^6^t, respectively; this accounted for 15.19%, 13.64%, and 11.45% of the total theoretical demand for P_2_O_5_ in southern China, respectively. Anhui Province, Sichuan Province, and Jiangxi Province had the highest theoretical demand for K_2_O, at 0.65×10^6^t, 0.65×10^6^ t, and 0.57×10^6^ t, respectively.

### 3.3 Straw returning to the field potential

#### 3.3.1 Nutrient substitution potential per unit sown area of straw returning to the field

**Figure 4:**
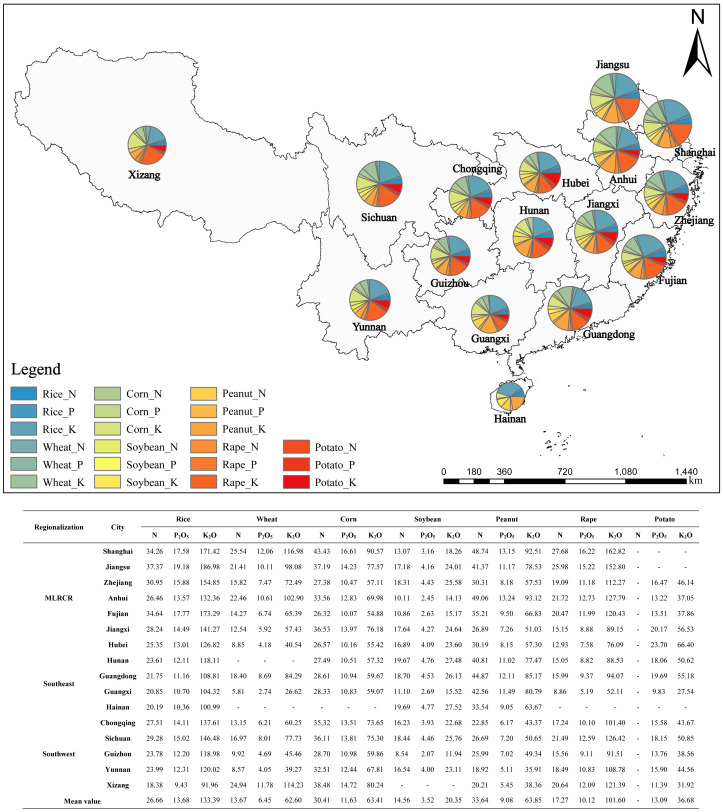
The replaceable amount of nutrients of main crops in straw returning to the field per unit sown area for cities in southern China in 2021 (kg/hm^2^). MLRCR (The middle and lower reaches of the Yangtze River).

By substituting the sown area of crops in southern China, the amount of straw nutrient resources, and the release rate of nutrients in the current season into Equations 6-8, the replaceable amount of nutrients of main crops in SRTTF per unit sown area for cities in southern China can be obtained (**Figure 3**). As shown in **Figure 4**, the replaceable amount of K_2_O (68.84 kg·hm^-2^) in straw returning from major crops in southern China was significantly higher than that of N (19.46 kg·hm^-2^) and P_2_O_5_ (9.65 kg·hm^-2^). The replacement potential of N, P_2_O_5_, and K_2_O for crop SRTTF in MLRCR was 22.00 kg·hm^-2^, 10.69 kg·hm^-2^, and 78.57 kg·hm^-2^, respectively. The substitution potential of N, P_2_O_5_, and K_2_O for crop SRTTF in MLRCR was higher than that in the southwestern and southeastern regions. The amount of substitutable N in SRTTF of the main crops in southern China was between 10.49 kg·hm^-2^ (Hainan Province) and 27.53 kg·hm^-2^ (Shanghai City); the amount of substitutable P_2_O_5_ was between 3.45 kg·hm^-2^ (Hainan Province) and 11.32 kg·hm^-2^ (Sichuan Province), and the amount of substitutable K_2_O was between 27.45 kg·hm^-2^ (Hainan Province) and 93.22 kg·hm^-2^ (Shanghai Province). The N, P_2_O_5_, and K_2_O fungible potential of rice, wheat, peanut, corn, and rape was highest in Shanghai, Jiangsu Province, Shanghai, the Tibet Autonomous Region, and Fujian Province; the N, P_2_O_5_, and K_2_O fungible potential of soybean and potato were highest in Hainan Province, Hunan Province, Guangdong Province, Jiangxi Province, and Hubei Province.

#### 3.3.2 Potential for straw returning to the field and fertilizer substitution

**Figure 5:**
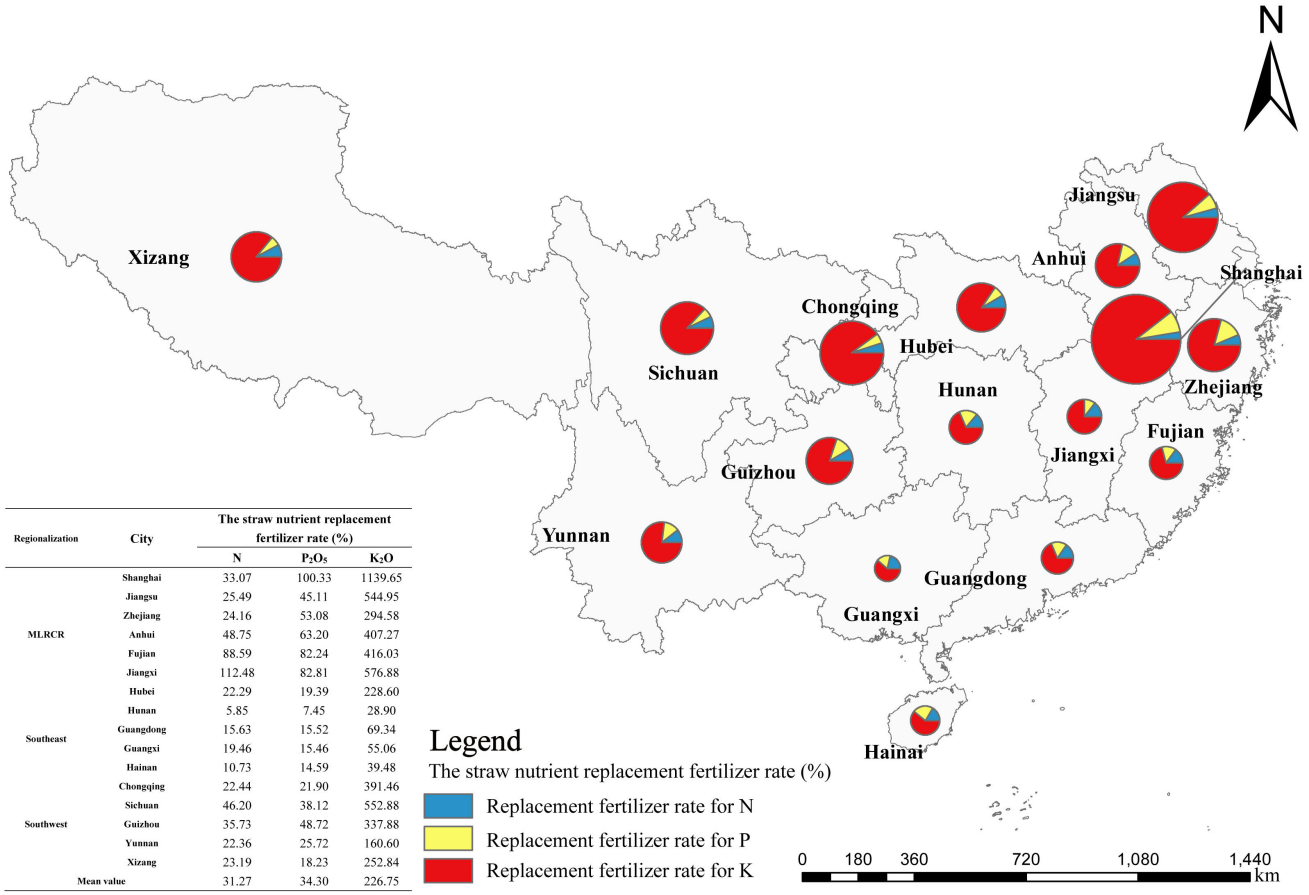
The theoretical straw nutrient replacement fertilizer rate of each province and autonomous region in southern China in 2021. MLRCR (The middle and lower reaches of the Yangtze River).

Based on the amount of N, phosphate, and potassium fertilizers in the National Statistical Yearbook data and the amount of straw nutrient resources (**Table 5**), the theoretical straw nutrient replacement fertilizer rate of each province and autonomous region in southern China in 2021 was estimated (**Figure 5**). The nutrient inputs of total SRTTF were 271.61×10^4^ t (N), 95.57×10^4^ t (P_2_O_5_), and 645.11×10^4^ t (K_2_O), which accounted for 31.27% (N), 34.30% (P_2_O_5_), and 226.75% (K_2_O) of the fertilizer used, respectively. The substitution rates of N, P_2_O_5_, and K_2_O were highest in MLRCR, at 50.69%, 63.74%, and 515.42%, respectively. The substitution rates of N, P_2_O_5_, and K_2_O were lowest in southeast China, at 12.91%, 13.25%, and 48.19%, respectively. The substitution rate of K_2_O in southern China varied, with the exception of the four provinces in southeastern China; the substitution rate of K_2_O in the other regions was greater than 100%, and the substitution rate of K_2_O in Shanghai was as high as 1,139.65%. K_2_O in straw in MLRCR and southwestern China can completely replace the input of K fertilizer.

**Table 5:** Classification table of straw returning methods.

First classification	Two-level classification	Explanations	References
Direct returning to the field	covering returning	After the crop is harvested, the crop straw directly crushed is directly used in the field by tossing and tilling or covering the surface	(Liu et al., 2021)
	high stubble returning	Cover whole stalks and high stubble in deep soil	(Qiao et al., 2020)
Indirect returning to the field	over-belly land returning	The straw is fed directly to livestock and then fertilized into the soil in the form of livestock manure	(Yang et al., 2019)
	composting land returning	The straw was thoroughly crushed and inoculated with a microbial agent for organic material decomposition, followed by its return to the field once the straw had undergone substantial decomposition	(Meng et al., 2022)
	biogas fertilizer returning	The straw is utilized as a feedstock for biogas production through fermentation treatment, resulting in the generation of both biogas and biogas slurry. Subsequently, the biogas slurry is applied to enrich the field soil	Sun (2017)
	fermentation land returning	After crushing, the straw is thoroughly blended with farm manure and stacked, followed by covering it with a plastic sheet to initiate pile fermentation. Subsequently, upon complete decomposition and fermentation of the straw, it is incorporated into the field soil	(Zhang et al., 2022)
	charcoal-based land returning	The crushed straw is subjected to anoxic combustion in a specialized carbonization furnace, devoid of open flame. Subsequently, the burned straw transforms into biogenic carbon particles, which can be directly applied to the field or processed with other fertilizers before being applied to the soil	(Zhu and Shui, 2023)

### 3.4 Analysis of straw nutrient return and utilization technology in different agricultural areas

The nutrient resources of straw in China are substantial. The use of appropriate straw nutrient return utilization technology is essential for maximizing the nutrient return potential, improving soil physical properties, increasing soil fertility levels, promoting soil carbon sequestration, reducing agricultural greenhouse gas emissions, optimizing the agricultural ecological environment, and achieving increases in crop yield. However, returning all straw resources available to the field not only fails to fertilize the soil and optimize the ecological environment of farmland but also leads to problems such as a low seed germination rate, low seedling emergence rate, and an increase in pests and diseases. Competition for N among seedlings has often been observed in the early stage of straw decomposition. China is a vast country with significant differences in climate, soil, crops, and economic conditions (Yang et al., 2010). The application of straw-returning technology based on local conditions is important for ensuring the efficient use of straw, improving soil structure, and promoting the sustainable and green development of agriculture. SRTTF is mainly performed via two methods: direct returning to the field (DR) and indirect returning to the field (IDR) (Bi et al., 2019; Zhao et al., 2021).

#### 3.4.1 Middle and Lower Reaches of the Yangtze River

The terrain of MLRCR is typical of a floodplain region. The high temperature and light in this region, sufficient heat, abundant rainfall, the concentrated distribution of rainfall, and the long frost-free period make this region optimal for the growth of rice (Tao, 2020); consequently, rice–wheat, rice–oil, and rice–rice rotations with multiple and efficient cropping patterns are often used. The Yangtze River floodplain is one of the most extensive multi-cropping areas in China (Zhou, 2020). The main characteristics of planting and straw returning in this area are as follows. (1) The soil has a heavy texture, the amount of straw is large, and straw burying is not effective. (2) The agricultural activities, including stubble use, are conducted over tight schedules, the rural labor force is insufficient, and the willingness to implement SRTTF is low. (3) The level of mechanization is high. (4) The use of land is highly intensive, the multiple cropping index is high, soil nutrient consumption is large, and the soil nitrogen utilization rate is low. (5) Soil microorganisms are active, and competition for N is strong (Liu, 2022; Zhou, 2020).

Because of the short agricultural cultivation time of the flat terrain, the high level of mechanization, and the abundant temperature and light, we suggest that the straw be returned directly to the field in MLRCR, which can reduce labor time through the use of mechanical equipment. Furthermore, the straw indirectly returned to the field can be used in areas with hills (**Table 5**).

#### 3.4.2 Southeast region

The southeast region has a tropical and subtropical monsoon climate, with an average annual temperature of 16.3 – 25.7°C and an average annual precipitation of 814.1 mm – 2,463.5 mm. The terrain is complex and undulating, and hills and plateaus are widespread. The abundant heat and water resources have created extremely favorable conditions for multi-crop rotations and an efficient farming system, including “rice–rice–rice,” “rice–rice–wheat,” “rice–rice–vegetable,” “rice–rice–vegetable,” “rice–rice–vegetable,” and “rice–rice–fallow” rotations in this area. The main characteristics of planting and straw returning in this area are as follows. (1) Soil degradation is severe, the amount of fertilizer required is large, the return to the field is not timely, and the actual content of nutrients is low. (2) Farming activities are conducted over tight schedules, costs are high, subsidies are insufficient, and the enthusiasm of farmers is not high. (3) The level of mechanization is low, the efficiency of agricultural machinery is low, the shredding effectiveness is low, and the wear of parts is severe, which results in decreases in the shredding length, impedes decomposition, and negatively affects the efficiency of SRTTF. (4) Planting and breeding are not coordinated, the level of feed is low, and the proportion of straw returned to the field is small. (5) Agricultural planning is not reasonable, and systematic research on the bearing capacity of livestock and poultry breeding is lacking; appropriate planning for animal husbandry is also lacking (Li, 2019).

We suggest that DR and IDR methods should be used in this region associatively because hills and plateaus are widespread and planting and breeding are not coordinated in the southeast (**Table 5**).

#### 3.4.3 Southwest region

The terrain in southwest China is high in the northwest and low in the southeast; this region is also characterized by complex and diverse landforms. Karst landforms are widely distributed, including mountains, plateaus, basins, hills, and plains. The climate is diverse in this region, and vertical variation in climate is significant. The dominant climate types from west to east include plateau climate, tropical seasonal rainforest climate, and subtropical monsoon climate (Ma et al., 2023). Many types of crops are planted in this area, and the planting systems are complex and diverse; the three dimensions of agriculture are readily apparent (Zhao, 2011). Due to the unique geographical environment and climate of this region, the main intensive farming rotations are “rice–oil,” “rice–vegetable,” and “vegetable–vegetable” rotations. The main characteristics of planting and straw returning in this area are as follows. (1) Karst rocky desertification is severe, the soil is barren, heavy applications of light organic fertilizer are used, and the fertilizer utilization rate is low. (2) Agricultural production is characterized by high consumption and low efficiency, the planting modes used are not capable of generating high-quality buckwheat, regional development is highly unbalanced, the links between operations are numerous, the labor supply is large, the labor intensity is high, income is low, management is intensive, and the enthusiasm of farmers to use SRTTF is low. (3) Engineering water shortages are prominent, and seasonal droughts are serious. (4) The level of agricultural mechanization is low, there is a lack of small machinery and equipment suitable for mountainous areas, the technical system used is not ideal, and there is a lack of technology appropriate for the decentralized management of straw utilization by farmers. (5) Although a policy that prohibits straw burning has been introduced, the long-term farming habits of farmers have resulted in a high straw burning rate, and the level of straw utilization is not high (Zhao, 2011).

The typical karst landform leads to serious rocky desertification and low mechanization level, so we suggest IDR as the main method in the southwest, and DR can be used in the dam area to improve agricultural production efficiency (**Table 5**).

## 4 Discussion

### 4.1 Straw Resources and Straw Returning to the Field Potential in Southern China

The results of this study indicate that the total amount of straw resources for major crops in southern China in 2021 is 3.35×10^8^ t, and the quantity of straw resources is substantial. The straw resources of three grain crops, rice, corn, and wheat, accounted for 83.94% of the total amount of straw resources for major crops in southern China. Among the three agricultural regions in southern China, MLRCR have the highest total straw resources, accounting for 63.60% of the total straw resources in the southern region.

A comparison of straw nutrient resources and crop nutrient requirements revealed that the content of N and P_2_O_5_ in straw in southern China was less than the crop demand for these two nutrients; that is, the total return of straw to the field in southern China is not sufficient for meeting the demand for N and P_2_O_5_ by crops. However, the amount of K_2_O resources in straw in all cities in southern China was much higher than the demand for this nutrient by crops. Therefore, the total return of straw to the field can meet the K_2_O required by crops. The quantities of N, P_2_O_5_, and K_2_O replaced by the straw of the main crops returned to the field per unit sown area in MLRCR were higher than those in the southwest and southeast regions. The substitutable amount of K_2_O was significantly higher than that of N and P_2_O_5_. Fertilizer substitution rates of N, P_2_O_5_, and K_2_O were highest in MLRCR, followed by the Southwest region and Southeast region. The fertilizer substitution rate of K_2_O (226.75%) was significantly higher than that of N (31.27%) and P_2_O_5_ (34.30%).

The results obtained by the three estimation methods were almost consistent. The nutrient replacement potential of straw was significantly higher in MLRCR than in the southeast and southwest regions, and the nutrient replacement potential of straw K_2_O was significantly higher than that of N and P_2_O_5_; these findings are consistent with the results of other studies. This might stem from significant differences in the tillage system, planting structure, climatic conditions, topography, and geomorphic environment among regions in southern China; the uneven spatial distribution of agricultural production areas and the pronounced regionalism in straw and nutrient resources might also contribute to explaining these differences. The MLRCR are plain areas with higher levels of mechanization than the other two agricultural areas. The climate conditions, including temperature and light, also promote agricultural development in the region.

### 4.2 Comparison of the methods for estimating the nutrient substitution potential of straw returning to the field

Many methods have been used to calculate the potential for SRTTF to be used as a substitute for chemical fertilizer.

The first involves estimating the amount of straw nutrient resources and the nutrient demand of crop growth and then subtracting the amount of straw nutrient resources from the demand for nutrients by crops. This method can be used to provide an initial understanding of whether SRTTF can meet the demand for nutrients by crops; it also provides a comprehensive understanding of differences in the demand for nutrients in different regions and by different crops. However, the nutrient release rates of N, P_2_O_5_, and K_2_O after SRTTF have not been studied, and the nutrient requirements of crops are affected by many factors, such as soil fertility, crop species, yield, and climatic conditions. No reliable and accurate method for estimating the nutrient requirements of crops on a large scale has been developed.

The second method involves estimating the seasonal release of straw nutrients per unit sown area, which considers the characteristics of nutrient release. The seasonal release rate of nutrients in this study was determined by a large number of experiments and studies, and these studies provide insights into soil fertility, crop varieties, irrigation conditions, and meteorological conditions of the main crop-producing areas.

Third, based on the amount of N, phosphate, and potassium fertilizer, combined with the amount of straw nutrient resources, the fertilizer substitution potential of straw N, P_2_O_5_, and K_2_O can be estimated using information on the actual amount of chemical fertilizer applied in each province, as this provides a better reflection of the current situation in each province.

In this study, three methods were used to estimate the SRTTF potential in southern China, and the results obtained by the three methods were almost consistent; however, some differences were observed.

The SRTTF potential obtained by subtracting the demand for nutrients by crops from the nutrients in straw indicates that the full amount of SRTTF can meet the demand for K_2_O by crops without the need for potassium fertilizer. These results were based on the seasonal release of straw nutrients per unit sown area, indicating that the substitution potential for K_2_O nutrients per unit sown area is 89.57 kg/hm^2^, 53.25 kg/hm^2^, and 67.69 kg/hm^2^ when straw is fully returned to the field in MLRCR, southeast regions, and southwest regions, respectively. The full amount of straw returned to the field is unable to meet the demand for K_2_O by crops, and additional potassium fertilizer needs to be applied. The amount of N fertilizer, phosphate fertilizer, and potassium fertilizer converted, along with the amount of nutrients in straw, indicates that the nutrients in straw can effectively replace the nutrients in fertilizer. The rate of full straw conversion to farmland that can replace chemical fertilizers was higher than 100% in cities in southern China, with the exception of the southeastern region, where the rate of full straw conversion to farmland that can replace chemical fertilizers was 48.19%; in Shanghai, the rate was as high as 1,139.65%.

The potential for SRTTF to be used as a substitute for chemical fertilizers varied significantly among the different calculation methods. This might stem from the fact that the different calculation methods take different factors into account. For example, some methods only consider the recommended fertilization amount per unit area of crops and the theoretical nutrient content of straw; others not only consider the theoretical nutrient resources of straw but also the seasonal release rate; and others perform calculations based on the amount of chemical fertilizer applied and the amount of straw resources. Each of the three methods has its advantages and disadvantages. However, this method is more appropriate for estimating the replaceable potential of straw because this method takes into account the current release rate of straw nutrients to the field and considers the process of decomposition, nutrient release, and the migration of straw after it is returned to the field. This method can also be used to ensure that the straw-returning approach used is optimal.

### 4.3 The main points for attention in the promotion of straw-returning technology in three agricultural areas

We have listed the main points that should be considered when promoting SRTTF. The SRTTF methods suitable for different agricultural areas should be adopted to achieve the efficient use of straw resources because of the wide variation in topography, climate, and agricultural planting patterns in different agricultural areas.

The following points merit attention in MLRCR to increase the SRTTF rate and improve the SRTTF effect. (1) In formulating policies, the government needs to encourage farmers to adopt direct straw-returning technology via different approaches. Secure long-term land rights are important for large-scale households, and this might be necessary for encouraging small farmers to adopt direct straw-returning technology via the implementation of policies, such as subsidies or penalties (Xu et al., 2018; Zhang, 2018). (2) Mechanical equipment is needed, such as harvesters with SRTTF machines, and the straw should be crushed once during crop harvesting. The straw-returning machine crushes the crop straw again; this is followed by plowing and mulching, and a rotary tiller is used to pulverize the soil twice to mediate its decomposition and promote the fertility of the soil over a large area (Li, 2011). (3) Mechanical improvements are needed. For example, the rotary tilling-stubble integrated sawtooth blade and the interaction of the straw-soil-rotary tilling knife should be optimized based on the mechanical properties of soil differing in straw content (Guo, 2017). (4) IDR to the field can mainly be used to accumulate waste in the field and add marsh fertilizer to the field. After straw compost retting and biogas production, the source of bacteria and insects decreases significantly. The biogas residue and biogas slurry produced after fermentation are good organic fertilizers; the use of these materials can promote the courtyard economy of farmers, improve the circulation of materials in agricultural ecosystems, and extend the ecological chain (Zhao et al., 2013).

The following points require attention in Southeast China to promote the SRTTF rate and improve the SRTTF effect. (1) Policy guidance should be strengthened, more publicity is needed, agricultural technicians require training, subsidies for SRTTF should be increased, and the enthusiasm of farmers for SRTTF should be promoted. (2) Animal husbandry in this area requires improvement and the agricultural layout needs to be planned rationally in areas with animal husbandry; over-belly straw-returning technology and direct straw-returning technology are the main approaches used to make full use of straw, manure, and fertilizer, as well as provide nutrients to the soil. (3) The policies of indirect straw returning technology should be strengthened, and fast rotting technology for straw should be popularized based on local conditions to improve the efficiency of straw nutrient returning to the field. (4) Investment in scientific research is needed, and the optimal system should comprise biochar, simple carbon technology, carbon-based fertilizers, and soil amendments.

The following points require attention in Southwest China to increase the SRTTF rate and improve the SRTTF effect. (1) In addition to increasing SRTTF subsidies, efforts to discourage straw burning behavior should be strengthened, more publicity is needed to make farmers aware of these policies, and technical training of farmers and the increased use of agricultural machinery by farmers are needed, which would enhance the ability of farmers to adopt straw returning (Wang and Yan, 2022). (2) The level of mechanization needs to be improved; the production of small harvesting, crushing, picking, and baling-supporting machinery in mountainous areas needs to be improved, the collection radius should be increased, the costs incurred by farmers should be reduced, the utilization of straw in hilly dam areas should be enhanced, and the utility of agricultural machinery and social services should be improved (Tong and Liu, 2018). (3) The introduction of commercial straw production enterprises and the establishment of straw collection and logistics systems are needed to promote the commercialization of straw, improve the straw processing capacity, and optimize the industrial structure, for example, through the use of straw as the base material to develop and produce edible fungi, the use of remaining waste (bacterial residue) for organic fertilizer, and SRTTF, as this will aid the development of the planting industry.

## 5 Conclusion

SRTTF can promote the sustainable development of agriculture and protect the environment as an important green agricultural technology in agricultural activities. Efficiently balancing chemical fertilizer consumption and SRTTF is crucial to the sustainable development of agriculture and the protection of the environment. We propose that the government should fully consider the geographical environment characteristics and local traditional farming habits when making policies to promote SRTTF and propose SRTTF technology suitable for local promotion, instead of simply banning straw burning, which is more conducive to promoting the implementation of straw returning technology.

## Author contributions

GM: Writing - review & editing; Methodology; Software. LX: Roles/Writing - original draft; Data curation; Resources. JZ: Supervision; Conceptualization; Project administration. All authors contributed to the article and approved the submitted version.
